# Supplementary material for: Assessing repetitive negative thinking in daily life: Development of an ecological momentary assessment paradigm
Source: PLoS One. 2020 Apr 20;15(4):e0231783. doi: 10.1371/journal.pone.0231783 (PMC7170251; doi:10.1371/journal.pone.0231783)
Supplement: S1 Table — (DOCX) [file pone.0231783.s001.docx]

**S1 Table. Multilevel Confirmatory Factor Analysis results for different exploratory hybrid models.**

|  | Exploratory hybrid models | χ² | *df* | CFI | SRMR | RMSEA | 90% CI_L_^1^ | 90% CI_U_^1^ |
| --- | --- | --- | --- | --- | --- | --- | --- | --- |
|  | FEEL, PROB, RPT, INTR | 709.780***  W: 605.96  B: 103.82 | 4 | .958 | .039 | .184 | .172 | .195 |
|  | FEEL, PROB, RPT, CTRL | 697.196***  W: 591.50  B: 105.69 | 4 | .961 | .038 | .182 | .171 | .193 |
|  | FEEL, PROB, RPT, BUR | 76.085***  W: 71.83  B: 4.25 | 4 | .996 | .011 | .059 | .048 | .071 |
|  | FEEL, PROB, INTR, CTRL | 814.203***  W: 702.49  B:111.71 | 4 | .952 | .043 | .197 | .185 | .208 |
|  | FEEL, PROB, INTR, BUR | 129.434***  W: 126.09  B: 3.34 | 4 | .993 | .015 | .077 | .066 | .089 |
|  | **FEEL, PROB, CTRL, BUR** | **50.096*****  **W: 40.57**  **B: 9.53** | **4** | **.997** | **.009** | **.047** | **.036** | **.059** |
| ^1^Upper/lower confidence interval for RMSEA; ***: p < .001; *: p < .05; n(within) = 10355, n(between) = 120. FEEL: feelings; PROB: problems; BUR: subjective burden; RPT: repetitiveness; INTR: intrusiveness; CTRL: uncontrollability; CFI: Comparative-Fit Index; SRMR: Standardized Mean Square Error of Approximation; RMSEA: Root Mean Square Error of Approximation; W: within-person level; B: between-person level. Final model is highlighted in bold. | | | | | | | | |
